# The association between loneliness and life satisfaction: examining spirituality, religiosity, and religious participation as moderators

**DOI:** 10.1186/s12877-023-04017-7

**Published:** 2023-05-16

**Authors:** T. Muhammad, Manacy Pai, K. Afsal, Priya Saravanakumar, C. V. Irshad

**Affiliations:** 1 Department of Family & Generations, International Institute for Population Sciences, Mumbai, 400088 India; 2 Department of Sociology and Criminology, Kent State University, Kent, OH 44242 USA; 3 Department of Migration & Urban Studies, International Institute for Population Sciences, Mumbai, 400088 India; 4 School of Nursing and Midwifery, Faculty of Health, University of Technology Sydney, Building 10, Level 7, 235 Jones St, Ultimo, NSW 2007 Australia; 5 School of Social Sciences and Languages, Vellore Institute of Technology, Vellore, 632014 India

**Keywords:** Perceived loneliness, Spirituality, Religiosity, Religious participation, Life satisfaction, Older adults, India

## Abstract

**Background:**

Future cohort of older adults may have to rely on non-family sources and forms of support, religion being one of them. This may be especially so, considering the recent longitudinal evidence that individuals are inclined to become more religious with increasing age. Thus, the purpose of the present study was to assess the association between loneliness and life satisfaction among older adults in India, and the extent to which the association between loneliness and life satisfaction is moderated by spirituality, religiosity, and religious participation.

**Methods:**

Data come from the Longitudinal Ageing Study in India, with a sample of 31,464 individuals aged 60 years and above. Multivariable logistic regression models were employed to examine the independent association of loneliness and life satisfaction. Further, an interaction analysis was conducted to examine the extent to which the association between perceived loneliness and life satisfaction is moderated by spirituality, religiosity and religious participation among older Indians.

**Results:**

The prevalence of low life satisfaction (LLS) was 30.84%; a total of 37.25% of participants reported feeling lonely, 12.54% reported a lack of spiritual experience, 21.24% reported not being religious, and 19.31% reported not participating in religious activities. Older adults who felt lonely had higher odds of LLS relative to peers who were not lonely. Further, the adverse impact of loneliness on LLS among older Indians is moderated by their spirituality, religiosity, and religious participation. Specifically, the adverse impact of loneliness on LLS was less negatively pronounced among older adults who were spiritual, religious, and engaged in religious activities.

**Conclusions:**

The study found an independent association between loneliness and lower life satisfaction among older adults in India. It also revealed that religiosity, spirituality and religious participation moderate the association between loneliness and lower life satisfaction. These findings, which underscore the health promoting benefits of religiosity and religious engagement, may be used to build on the interaction between religious and faith-based groups and public health professionals.

## Background

Extensive research exists on life satisfaction in older ages. And given the rapidly aging world population and the social, economic, and health challenges that accompany this demographic shift, research on later life wellbeing continues to gain traction [1, 2]. There is the view that increasing age is associated with a sense of maturity and self-actualization, which may help older adults adapt to the challenges associated with normal aging [3, 4]. Empirical research finds that older adults who selectively optimize available opportunities to participate socially are able to maximize gains and minimize losses, despite the changes of later life [2, 5–7]. For instance, retired older adults who volunteer report higher life satisfaction compared to retirees who are not volunteers [8, 9]. Likewise, older adults who make new friends, learn new skills, and pursue leisure activities report better subjective wellbeing relative to their less socially engaged peers [10]. Alternatively, some research finds reduced subjective wellbeing, including life satisfaction among older adults [11]. Growing old is associated with loss of work, shrinking social networks, children growing up and moving away, and risk of multiple health conditions, which may lead to mobility limitations. Under these conditions, which are associated with normal aging, it is only natural for older adults to feel socially irrelevant, isolated, and lonely [12, 13].

That said, not individuals who are socially isolated feel lonely and not all who feel lonely are socially isolated. Social isolation is the objective and structural measure of the absence of social connections and perhaps a lack of social engagement [14–16] Alternatively, loneliness represents the subjective experience or perception of being isolated [14–16]. Though socially isolated older adults often report feeling lonely, social isolation and loneliness are not always strongly correlated [15–17]. Loneliness, however, is a serious public health risk impacting a sizeable portion of older adults globally [18–20]. It is an important indicator of psychosocial wellbeing given its marked and often adverse, association with countless mental and physical health outcomes, including depression, anxiety, immobility, hypertension, heart disease, weakened immunity, inflammation, cognitive decline, dementia, and mortality [19, 21–23]. As such, among older adults, one of the most essential indicators of life satisfaction is loneliness.

Although the risk of loneliness is likely to surge with age, clearly not all older adults are lonely [24]. The question then remains: What renders some older adults to be more resilient to loneliness while their peers remain susceptible to it? Among other factors, research suggests that aspects related to religion may be of significance for wellbeing, especially later in life [25–27]. Worship services, bible reading, prosocial activities that religious institutions often partake in, and social gatherings (e.g., communal meals; holiday celebrations, etc.) may help cultivate a sense of belonging, friendships and in consequence, social support, and cohesion. Religious attendance, in fact, is connected with wider social networks and more regular interactions with network members [28–30]; and older adults who report religious attendance also tend to report higher life satisfaction [31].

In addition to religious attendance, spirituality, and religiosity also are found to positively predict subjective wellbeing [32, 33]. The feeling of being part of something larger than the self, in sync with a higher power, and connected to the universe may facilitate positive affect and in turn, a positive appraisal of life and one’s place in it [34, 35]. Practices – such as prayer, spiritually guided meditation, yoga, and nature walks – are found to foster feelings of peace, hopefulness, gratitude and forgiveness [36–38], all of which can improve social connectedness, reduce loneliness, and in turn, promote life satisfaction. Spirituality and religiosity both also are found to elevate one’s sense of self-esteem (Lim & Putnam, 2010), which is a robust predictor of health, including life satisfaction [1, 2, 39]. To the extent that religious attendance, religiosity, and spirituality facilitate social, emotional, and psychological resources, each of them are likely to temper the adverse impact of loneliness on life satisfaction among older adults.

Although various studies have documented the link between spirituality, religiousness, religious participation and life satisfaction, few focus on older individuals and especially so, in lower and middle countries (LMICs), like India. The number of older adults aged 65 years has crossed 700 million, it will rise twofold to 1.5 billion in 2050 [40], and two-thirds of this aging population will reside in LMICs, like India. India is a collectivistic society with differential experiences and expectations related to social relationships and social engagements, both of which are characteristic of the customary kin-centric care and support [41]. Given this, the significance of religiosity, religious participation, and even spirituality for health and wellbeing may vary from what we observe in studies limited to western nations. Further, while Indian older adults, given filial piety, are revered and cared for by family, the traditional sources of social and emotional support (namely, adult children) cannot be taken for granted due to evolving family structures, declining fertility, perceived rise in individualistic attitudes, and household nuclearization [42, 43]. Future cohort of older adults may have to rely on non-family sources and forms of support, religion being one of them. This may be especially so considering the recent longitudinal evidence that individuals are inclined to become more religious with increasing age [44, 45].

Given the complexity associated with the constructs of religiosity and spirituality it is important to clarify the difference between the two. Religiosity often is deemed as the formal declaration or assertion of one’s beliefs about and relationship with God or the sacred. It typically is actualized within the context of organized religion [46, 47]. Conversely, spirituality, which can be practiced in or outside the confines of a religious context involves the pursuit for meaning in life and consequently, those who are spiritual typically are aiming for transcendence, self-actualization, and togetherness [48, 49]. Additionally, religious participation, which also is of interest in the present study, refers to the actual engagement in religious activities or activities, such as praying rituals, prayer groups, daily devotion, and religious gatherings, including religious retreats [29, 50, 51].

**Fig. 1:**
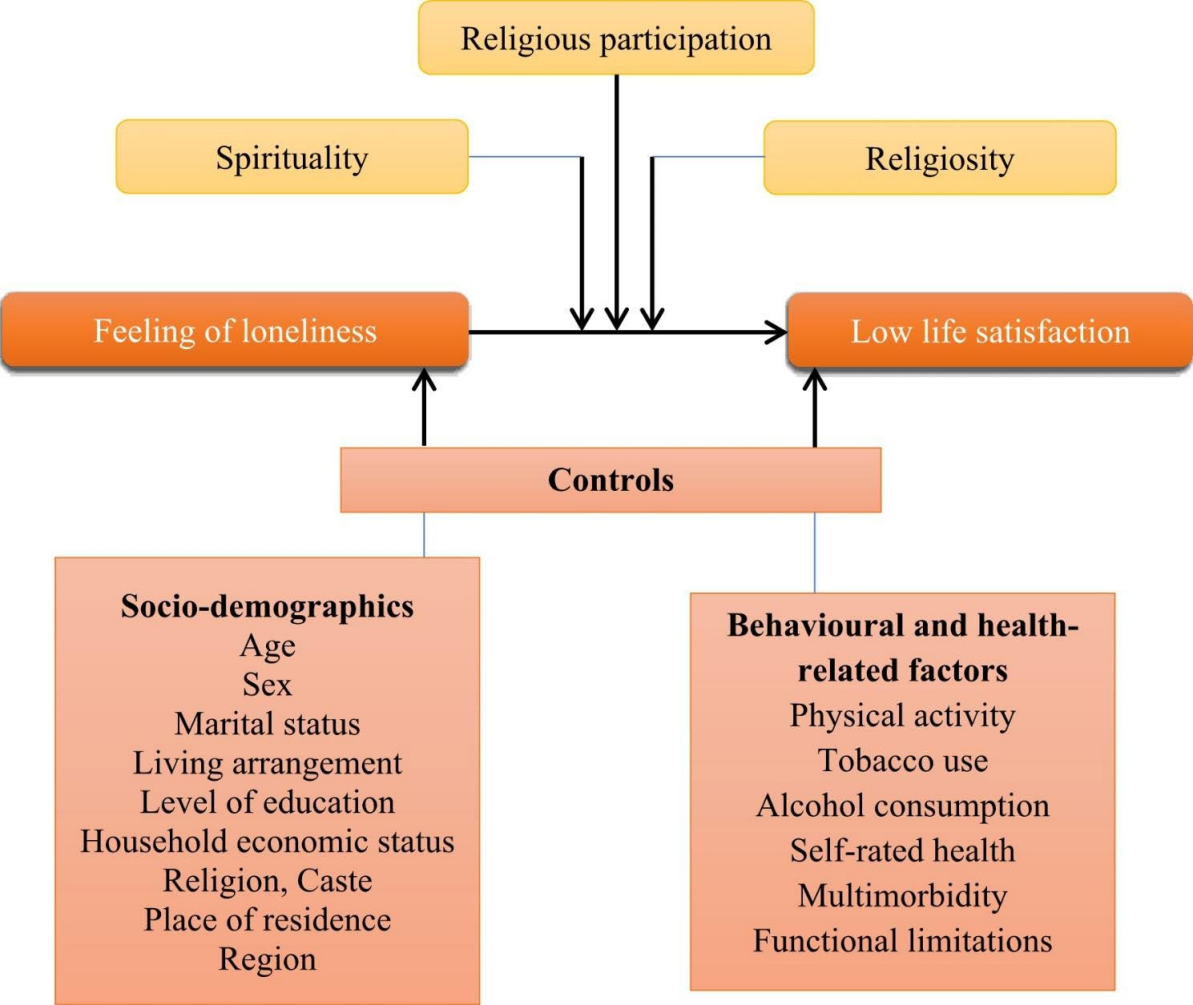
Conceptual model

As such, the purpose of the present study is to assess (1) the association between loneliness and life satisfaction among older adults in India; and (2) the extent to which the association between loneliness and life satisfaction is moderated by (a) spirituality, (b) religiosity, and (c) religious participation. The conceptual framework is shown in Fig. 1.

## Methods

### Study design and sample

Data come from wave 1 of the Longitudinal Aging Study in India (LASI), which was conducted during 2017-18. LASI, which is the Indian version of the Health and Retirement Study (HRS), is a nationally representative survey conducted by the International Institute for Population Sciences (IIPS) in collaboration with the Harvard T.H. Chan School of Public Health and the University of Southern California (USC). LASI is India’s first nationwide longitudinal aging study designed to investigate the health, economic, social, and psychological aspects of population aging. Detailed information on the survey design, sampling frame, and data collection is available in the LASI wave-1 Report [52]. LASI included 72,250 adults aged 45 and above including their spouses across all 30 states and 6 union territories of India as per the 2011 Indian Census. The survey adopted a multistage stratified cluster sampling design to follow the sample biennially for 25 years. The total sample for the present study comprised of 31, 464 older Indians aged 60 and above, among which 15,098 were men and 16,366 were women.

### Measures

#### Outcome variable

Life satisfaction among older adults was assessed using the following five questions (a) In most ways, my life is close to ideal; (b) The conditions of my life are excellent; (c) I am satisfied with my life; (d) So far, I have got the important things I want in life; and (e). If I could live my life again, I would change almost nothing. The responses of “strongly disagree,” “somewhat disagree,” “slightly disagree,” “neither agree nor disagree,” “slightly agree,” “somewhat agree,” and “strongly agree” were recorded using a 7-point Likert scale. Further, a composite score was generated by summing all the items with a score ranging from 5 to 35 (higher score representing higher life satisfaction) and Cronbach’s alpha of 0.90. Relying on previous studies [53], the scale subsequently was dichotomized based on a cut-off point of 20 and recoded into 0 ‘no’ (21–35) and 1 ‘yes’ (5–20) representing low life satisfaction (LLS).

### Main explanatory variables

***Loneliness***: The LASI questionnaire does not contain a well-recognized UCLA three-item loneliness scale. Therefore, to capture the feeling of loneliness, a single-item measure was used from the 10-item Centre for Epidemiological Studies Depression Scale (CES-D-10) [54–56]. A question was asked to the respondents: During the past week, how often do you feel alone? The responses were 1, rarely or never (less than 1 day); 2, sometimes (1 or 2 days); 3, often (3 or 4 days); and 4, most or all of the time (5–7 days). Based on responses, we constructed a binary variable where 1 is coded as “lonely” (Sometimes/Often/Most or all of the time) and the remaining categories coded as 0 “not lonely” [57].

***Daily spiritual experiences***: To measure spirituality, we relied on the daily spiritual experience scale (DSES), which originally was developed for use in health studies [58, 59], and validated and adapted cross-culturally [60, 61]. The variable, which is generated using 4 items available in LASI [52] did not have the word “God.” This is because in India, a substantial proportion of the population worships innumerable gods representing either a philosophy, a natural power or a representation of a certain moral value or quality [62]. The four items (Cronbach’s alpha: 0.89) assessed the experiences, rather than particular beliefs or behaviors, in order to transcend the boundaries of any religion in particular [58]. Those who reported any of the four experiences were coded as “Yes” i.e., experiencing spirituality once or several times a day and those without any of the daily spiritual experiences were coded as “No” i.e., did not experience spirituality at least once in a day.

#### Religiosity

Religiosity is assessed using the question ‘How important would you say religion is in your life? And the responses include very important, somewhat important, and not too important. This single-item subjective report was validated in measuring religious/spiritual importance ranking highest among a diverse latent factor structure [63]. It was recoded as 0 ‘no’ (not too important) and 1 ‘yes’ (very/somewhat important).

#### Religious participation

Religious participation is assessed using the question ‘How often do you engage in the following religious activities? (a) done pooja or prayer? (b) attended religious services (at temple/mosque/church, etc.)? and (c) involved in *satsang/bhajan/ kirtan*/any religious gathering? The responses include every day, more than once a week, once a week, 1 to 3 times a month, 1 or more times a year and not at all. We used these responses to recode less than once a month or 0 as “no religious participation” and at least once a month or 1 as “yes, religious participation.”

### Socio-demographic characteristics

Age was categorized into age groups of 60–69 years, 70–79 years, and 80 + years. Sex was coded as male and female. Marital status was coded as currently married and unmarried. Unmarried included widowed/divorced/separated/never married. Types of living arrangements were grouped into ‘living alone’, ‘living with spouse’ and ‘other living arrangements.’ Educational status was coded as no education/primary, secondary, and higher. Working status was coded as never worked, currently not working, working, and retired.

### Behavioral factors

Physical activity was categorized as yes (every day, more than once a week, once a week, one to three times in a month), and no (hardly ever or never). The following question was used to measure physical activity “How often do you take part in sports or vigorous activities, such as running or jogging, swimming, going to a health center or gym, cycling, or digging with a spade or shovel, heavy lifting, chopping, farm work, fast bicycling, cycling with loads”? Tobacco and alcohol consumption were coded “No” for individuals who have never engaged in substance use and ”Yes” representing their counterparts with history of substance use.

### Health-related factors

Religious participation, be it worship services or other social activities, requires travel to another location, which may require certain level of functional health [64]. For instance, physical disability, cancer, stroke, and other debilitating illnesses have shown to compromise religious participation among older adults [65–67]. To that end, we control for several health related variables. Self-rated health (SRH) was coded such that “good” indicates the perception of being in excellent, very good, and good health, whereas “poor” was indicative of fair and poor condition of one’s health. Multimorbidity refers to the presence of two or more chronic diseases which include hypertension, chronic heart disease, stroke, any chronic lung disease, diabetes, cancer or malignant tumor, any bone/joint disease and neurological/ psychiatric diseases. It was assessed by interviewer’s question whether the respondent has ever been diagnosed with the above mentioned diseases by a doctor or a health professional. Reporting two or more diseases was considered as multimorbid and otherwise no [68]. The ADL (activities of daily living) and IADL (instrumental activities of daily living) functioning were coded as high and low; “high” representing the absence of any functional disability and “low” representing a disability [69].

### Household/community factors

Based on recommendations for “better” indicators of Socio-economic Status (SES) in LMICs [70], older adults’ SES is assessed using the monthly per-capita consumption expenditure (MPCE) quintile. Sets of 11 and 29 questions on the expenditures on food and non-food items, respectively, were used to canvass the sample households. Food expenditure was collected based on a reference period of seven days, while the non-food expenditure was collected using reference periods of 30 days and 365 days [52]. Food and non-food expenditures have been standardized to a 30-day reference period. The variable was divided into five quintiles i.e., from poorest to richest.

Religion was recoded as Hindu, Muslim, and Others. Given the caste based variations in the association between SES and health and mortality [71, 72], we also included respondents’ self-reported caste, and was coded as Scheduled Caste/ Scheduled Tribe (SC/ST), Other Backward Class (OBC), and others. Lastly, the geographical region is coded as north, central, east, northeast, west, and south; and place of residence was classified into urban vs. rural areas.

### Statistical analysis

We conducted descriptive statistics and bivariate analysis to assess the prevalence of LLS along with explanatory variables. Chi-Square test was used to examine the significance of possible associations among variables and p-values were reported. Further, multivariable logistic regression models were used to test the hypotheses of the study. The results are presented in the form of crude (Crude OR) and adjusted odds ratio (Adjusted OR) with a 95% confidence interval (CI). Individual weights were used to make the estimates nationally representative. All the analyses were conducted using STATA version 15.1.

Along with an unadjusted model, the multivariable analysis provides two models to explain the adjusted estimates. Model-1 provides the estimates of LLS adjusted for the selected socio-demographic variables. Model-2 is additionally adjusted for the selected health and behavioral factors. Also, an interaction analysis was conducted to examine the extent to which the association between perceived loneliness and LLS is moderated by spirituality, religiosity and religious participation among older Indians.

During the multivariable analysis, the observations with missing information in any of the study variables (n = 1287) were dropped and the final study sample was 30,177 older adults. The socio-demographic characteristics of the included and excluded samples were compared. We observed no statistically significant differences in the two samples, suggesting no potential impact of missingness in the current analyses.

## Results

### Socioeconomic and health profile of older adults

Table 1 represents the socio-economic and health profile of older adults. A total of 37.25% of the study participants reported feeling lonely. Only 12.54% of older participants reported having no daily spiritual experiences. The proportion of older adults who reported religion as not important to them was 21.24%, whereas, 19.31% of the respondents did not participate in religious activities. The share of the sample in the age group of 80 years and above in the study was 11.29%. In the study sample, 52.55% of older adults were females. A total of 74.02% were either not educated at all or had a primary level education, whereas, only 7.74% were highly educated. 29.45% of the study participants belonged to urban areas while 70.55% lived in rural India.

**Table 1:** Socio-demographic and health characteristics of study participants (n = 31,464)

Variables		Number of missing cases	Sample percentage (%)	Number of participants
**Age (in years)**		-		
60–69			58.51	18,974
70–79			30.20	9,101
80+			11.29	3,389
**Sex**		-		
Male			47.45	15,098
Female			52.55	16,366
**Marital status**		-		
Currently in union			61.63	19,920
Not in union			38.37	11,544
**Living arrangement**		-		
Alone			5.68	1,622
With spouse			20.33	6,215
Others			73.99	23,627
**Level of education**		-		
No/primary			74.02	22,729
Secondary			18.24	6,106
Higher			7.74	2,629
**Work status**		-		
Never worked			26.43	8,784
Not working			36.45	10,990
Working			29.87	8,997
Retired			7.25	2,693
**Spirituality**		1080		
No			12.54	3,574
Yes			87.46	26,810
**Religiosity**		572		
No			21.24	6,068
Yes			78.76	24,824
**Religious participation**		54		
No			19.31	5,140
Yes			80.69	26,270
**Physical activity**		266		
No			68.90	21,653
Yes			31.10	9,545
**Tobacco use**		252		
No			59.83	19,034
Yes			40.17	12,178
**Alcohol consumption**		245		
No			85.41	25,855
Yes			14.59	5,364
**SRH**		666		
Good			75.79	23,685
Poor			24.21	7,113
**Multimorbid**		91		
No			75.95	23,576
Yes			24.05	7,797
**ADL functioning**		128		
High			76.23	24,642
Low			23.77	6,694
**IADL functioning**		169		
High			51.64	17,449
Low			48.36	13,846
**Loneliness**		1070		
No			62.75	19,399
Yes			37.25	10,995
**MPCE quintile**		-		
Poorest			21.7	6,484
Poorer			21.71	6,477
Middle			20.95	6,416
Richer			19.19	6,170
Richest			16.45	5,917
**Religion**		-		
Hindu			82.22	23,037
Muslim			11.28	3,731
Others			6.50	4,696
**Caste**		-		
SC/ST			27.03	10,313
OBC			45.23	11,886
Others			27.74	9,265
**Place of residence**		-		
Urban			29.45	10,739
Rural			70.55	20,725
**Region**		-		
North			12.59	5,812
Central			20.95	4,262
East			23.64	5,757
Northeast			2.97	3,752
South			22.68	7,578
West			17.17	4,303

Counts are un-weighted and percentages are weighted; SRH: Self-Rated Health; ADL: Activities of daily living; IADL: Instrumental activities of daily living; MPCE: Monthly per capita consumption expenditure; SC/ST: Scheduled caste/scheduled tribe; OBC: Other backward classes

### Bivariate analyses of LLS among older indian adults

Table 2 depicts the share of older adults who are dissatisfied with their lives (LLS). The prevalence of LLS in the current sample was 30.84%. About 40.86% of older adults who felt lonely also were dissatisfied with their lives compared to 26.94% of those who did not report loneliness. Alternatively, 29.18% and 29.38% of older adults who had spiritual experiences and were religious were dissatisfied with their lives compared to 52.62% and 39.18% of older adults with no spiritual experience and no religiosity, respectively. Similarly, only 29.21% of older adults who participated in religious activities were dissatisfied with their lives relative to 37.55% of those who did not participate in such activities. Older women had a substantially higher level of dissatisfaction (32.54%) than older men (28.96%).

**Table 2:** Bivariate analysis of low life satisfaction (LLS) among older adults

Variables	Percentage (%)	p-values
**Loneliness**		< 0.001
No	26.94	
Yes	40.86	
**Spirituality**		< 0.001
Yes	52.62	
No	29.18	
**Religiosity**		< 0.001
Yes	39.18	
No	29.38	
**Religious participation**		< 0.001
No	37.55	
Yes	29.21	
**Age (in years)**		0.324
60–69	30.88	
70–79	31.05	
80+	30.1	
**Sex**		< 0.001
Male	28.96	
Female	32.54	
**Marital status**		< 0.001
Currently in union	28.59	
Not in union	34.47	
**Living arrangement**		< 0.001
Alone	45.89	
With spouse	29.59	
With others	30.03	
**Level of education**		< 0.001
No/primary	34.68	
Secondary	21.03	
Higher	17.27	
**Work status**		< 0.001
Never worked	30.83	
Not working	32.85	
Working	31.81	
Retired	16.84	
**Physical activity**		0.018
No	30.88	
Yes	31.83	
**Tobacco use**		< 0.001
No	29.77	
Yes	33.28	
**Alcohol consumption**		< 0.001
No	30.83	
Yes	33.24	
**SRH**		< 0.001
Good	28.22	
Poor	42.47	
**Multimorbid**		0.001
No	31.44	
Yes	29.68	
**ADL**		< 0.001
High	30.15	
Low	34.08	
**IADL**		< 0.001
High	28.72	
Low	33.65	
**MPCE quintile**		< 0.001
Poorest	36.93	
Poorer	32.77	
Middle	28.73	
Richer	27.26	
Richest	27.15	
**Religion**		< 0.001
Hindu	37.23	
Muslim	30.26	
Others	25.58	
**Caste**		< 0.001
SC/ST	31.09	
OBC	29.77	
Others	29.65	
**Place of residence**		< 0.001
Urban	25.16	
Rural	33.22	
**Region**		< 0.001
North	33.02	
Central	31.32	
East	35.34	
Northeast	25.24	
West	36.85	
South	15.52	
**Total**	30.84	

p-values are based on Chi-Square test; ADL: Activities of daily living; IADL: Instrumental activities of daily living; MPCE: Monthly per capita consumption expenditure; SC/ST: Scheduled caste/scheduled tribe; OBC: Other backward classes

### Multivariable regression estimates of LLS among older adults

In the multivariable logistic regression analysis (Table 3), we find that older adults who felt lonely had 92% significantly higher odds of LLS [Crude OR: 1.92, CI: 1.64–2.25] in comparison to their peers who were not lonely. The association remains same after adjusting for all the socioeconomic and health-related variables in the study [Adjusted OR: 1.61, CI: 1.46–1.77]. Older adults who reported daily spiritual experiences [Crude OR: 0.37, CI: 0.33–0.42] and were religious [Crude OR: 0.65, CI: 0.57–0.74] reported lower odds of having LLS than their counterparts. While slight attenuation is observed, the association remains same after adjusting for all the covariates for spirituality [Adjusted OR: 0.43, CI: 0.38–0.49] and religiosity [Adjusted OR: 0.83, CI: 0.74–0.97]. Compared to older adults who did not participate in religious activities, those who engaged in religious activities after adjusting for other variables had lower odds of LLS [Adjusted OR: 0.84, CI: 0.75–0.95] in the current study.

**Table 3:** Multivariable logistic regression estimates of low life satisfaction (LLS) with socioeconomic and health characteristics among older adults in India

Variables		Crude OR (95% CI)	Adjusted OR (95% CI) Model 1	Adjusted OR (95% CI) Model 2
**Loneliness**	No	Ref.	Ref.	Ref.
	Yes	1.92*** (1.64–2.25)	1.68*** (1.53–1.85)	1.61*** (1.46–1.77)
**Spirituality**	No	Ref.	Ref.	Ref.
	Yes	0.37*** (0.33–0.42)	0.43*** (0.38–0.49)	0.43*** (0.37–0.49)
**Religiosity**	No	Ref.	Ref.	Ref.
	Yes	0.65*** (0.57–0.74)	0.84*** (0.74–0.95)	0.83*** (0.74–0.94)
**Religious participation**	No	Ref.	Ref.	Ref.
	Yes	0.80*** (0.73–0.90)	0.81*** (0.72–0.92)	0.84*** (0.75–0.95)
**Age (in years)**	60–69		Ref.	Ref.
	70–79		0.98 (0.88–1.09)	0.96 (0.86–1.07)
	80+		0.88 (0.75–1.04)	0.82** (0.70–0.98)
**Sex**	Male		Ref.	Ref.
	Female		0.96 (0.87–1.07)	1.03 (0.92–1.15)
**Marital status**	Currently in union		Ref.	Ref.
	Not in union		1.06 (0.94–1.18)	1.07 (0.96–1.20)
**Living arrangement**	Alone		Ref.	Ref.
	With spouse		0.68*** (0.54–0.85)	0.69*** (0.55–0.86)
	Others		0.65*** (0.53–0.79)	0.66*** (0.54–0.80)
**Level of education**	No education		Ref.	Ref.
	Primary		0.61*** (0.52–0.72)	0.63*** (0.53–0.73)
	Secondary/higher		0.60*** (0.47–0.78)	0.66*** (0.51–0.84)
**Work status**	Never worked		Ref.	Ref.
	Not working		1.13* (0.99–1.30)	1.08 (0.95–1.23)
	Working		1.05 (0.91–1.21)	0.99 (0.86–1.15)
	Retired		0.71*** (0.56–0.91)	0.68*** (0.54–0.86)
**Physical activity**	No			Ref.
	Yes			1.20*** (1.08–1.35)
**Tobacco use**	No			Ref.
	Yes			1.17*** (1.06–1.28)
**Alcohol consumption**	No			Ref.
	Yes			1.03 (0.91–1.17)
**SRH**	Good			Ref.
	Poor			1.57*** (1.41–1.75)
**Multimorbid**	No			Ref.
	Yes			0.95 (0.84–1.07)
**ADL**	High			Ref.
	Low			1.23*** (1.09–1.38)
**IADL**	High			Ref.
	Low			0.96 (0.87–1.06)
**MPCE quintile**	Poorest		Ref.	Ref.
	Poorer		0.93 (0.82–1.06)	0.94 (0.82–1.07)
	Middle		0.79*** (0.69–0.90)	0.80*** (0.70–0.91)
	Richer		0.72*** (0.63–0.84)	0.73*** (0.63–0.84)
	Richest		0.76*** (0.64–0.89)	0.76*** (0.65–0.89)
**Religion**	Hindu		Ref.	Ref.
	Muslim		0.76*** (0.68–0.85)	0.77*** (0.69–0.86)
	Others		0.77*** (0.68–0.87)	0.78*** (0.69–0.89)
**Caste**	SC/ST		Ref.	Ref.
	OBC		1.13* (0.99–1.28)	1.11 (0.98–1.26)
	Others		0.89 (0.76–1.03)	0.88* (0.75–1.02)
**Place of residence**	Urban		Ref.	Ref.
	Rural		1.06 (0.94–1.19)	1.02 (0.91–1.15)
**Region**	North		Ref.	Ref.
	Central		0.84*** (0.74–0.96)	0.81*** (0.72–0.92)
	East		1.04 (0.93–1.17)	0.96 (0.86–1.08)
	Northeast		0.72*** (0.62–0.84)	0.68*** (0.58–0.79)
	West		1.21*** (1.06–1.38)	1.15** (1.00–1.32)
	South		0.38*** (0.33–0.45)	0.36*** (0.31–0.42)
Pseudo R-Square			0.0791	0.0880
Observations (n)		30,177	30,177	30,177

*if p < 0.05, **if p < 0.01, ***if p < 0.001; Crude OR: Unadjusted Odds Ratio; Adjusted OR: Adjusted Odds Ratio; Model 1 is adjusted for individual socio-demographic factors (age, education and marital status, along with household factors (wealth quintile, religion, caste, place of residence, region); Model 3 is additionally adjusted for behavioural factors (physical activity, tobacco and alcohol use), and health-related factors (SRH, multimorbidity and ADL and IADL functioning)

### Interaction of loneliness with spirituality, religiosity and religious participation on LLS among older adults

Table 4 presents the interaction of loneliness with spirituality, religiosity and religious participation of older individuals on their LLS. Older participants who reported no daily spiritual experiences had higher odds of LLS [Adjusted OR: 1.53, CI: 1.07–2.20] whereas, those who felt lonely and reported some daily spiritual experiences [Adjusted OR: 0.68, CI: 0.55–0.84] reported lower odds of LLS. Older adults who felt lonely and were not religious were 1.51 times [Adjusted OR: 1.51, CI: 1.23–1.87] more likely to have LLS whereas, those who were lonely and were religious were 1.32 times [Adjusted OR: 1.32, CI: 1.12–1.55] more likely to have LLS than those who did not feel lonely and not religious. At the same time, older adults who were lonely and did not participate in religious activities had 1.48 higher odds [Adjusted OR: 1.48, CI: 1.16–1.89] of LLS, whereas, those older adults who were lonely and participated in religious activities were 37% significantly more likely [Adjusted OR: 1.37, CI: 1.15–1.63] to have LLS relative to those who were lonely and did not participate in religious activities.

**Table 4:** Interaction of loneliness with spirituality, religiosity and religious participation on low life satisfaction (LLS) among older adults

Variables	Adjusted OR (95% CI) Model 1	Adjusted OR (95% CI) Model 2	Adjusted OR (95% CI) Model 3
**Loneliness X Spirituality**			
No X No	Ref.		
No X Yes	0.42*** (0.36–0.49)		
Yes X No	1.53** (1.07–2.20)		
Yes X Yes	0.68*** (0.55–0.84)		
**Loneliness X Religiosity**			
No X No		Ref.	
No X Yes		0.80*** (0.69–0.93)	
Yes X No		1.51*** (1.23–1.87)	
Yes X Yes		1.32*** (1.12–1.55)	
**Loneliness X Religious participation**			
No X No			Ref.
No X Yes			0.86* (0.73–1.01)
Yes X No			1.66*** (1.36–2.04)
Yes X Yes			1.37*** (1.15–1.63)
Pseudo R-Square	0.0883	0.0897	0.0881
Observations	30,177	30,177	30,177

*if p < 0.05, **if p < 0.01, ***if p < 0.001; Adjusted OR: Odds Ratio Adjusted for all socio-demographic, behavioural and health-related factors

**Fig. 2:**
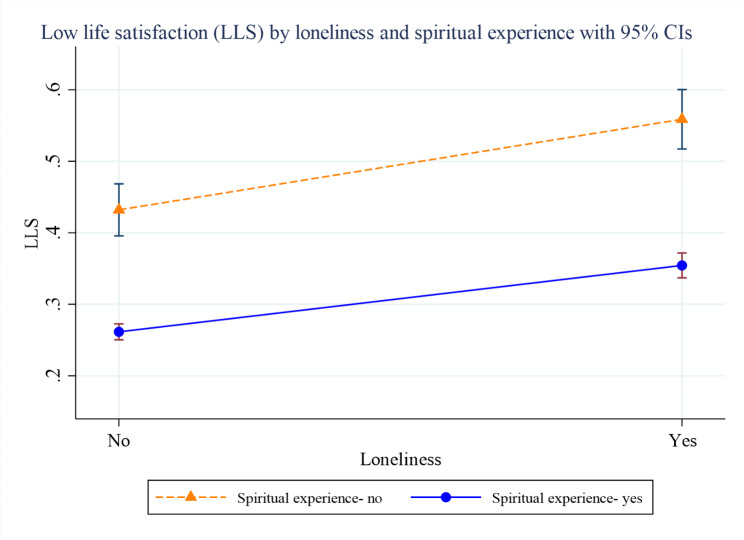
Margins plot of the interaction of spiritual experience with loneliness on low life satisfaction (LLS) among older adults (p-value = 0.498)

**Fig. 3:**
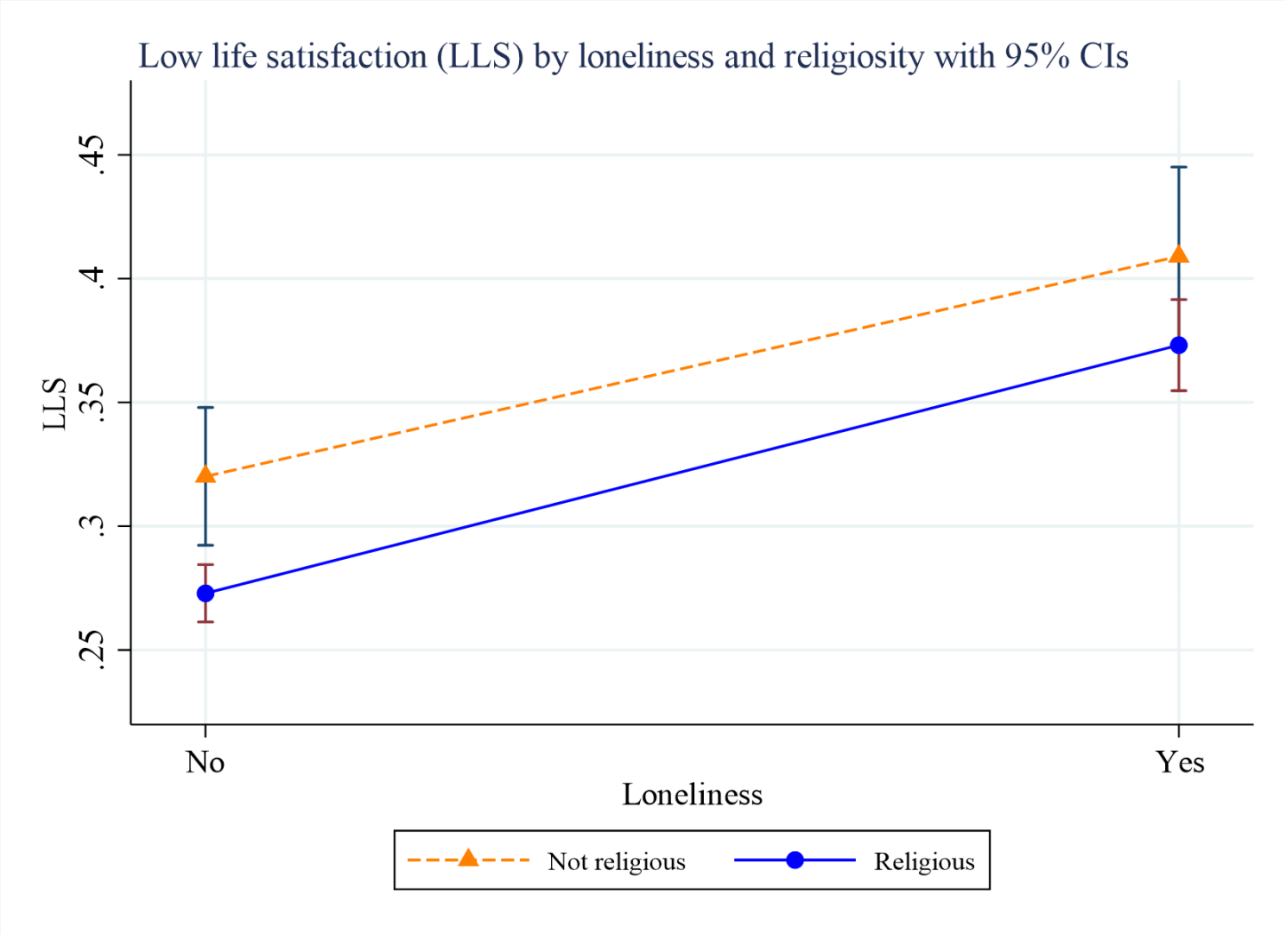
Margins plot of the interaction of religiosity with loneliness on low life satisfaction (LLS) among older adults (p-value = 0.365)

**Fig. 4:**
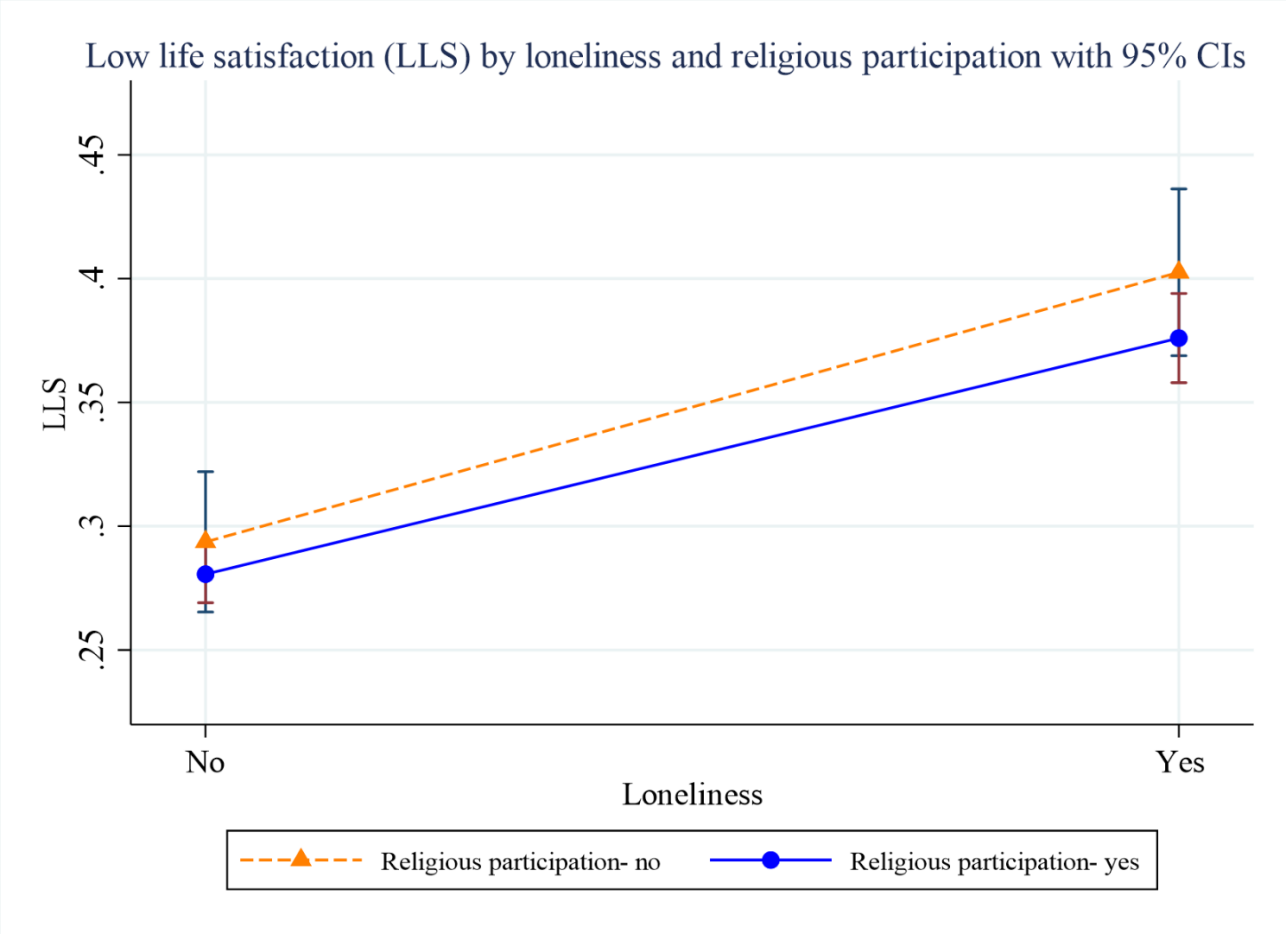
Margins plot of the interaction of religious participation with loneliness on low life satisfaction (LLS) among older adults (p-value = 0.707)

Figures 2, 3 and 4 present the margins plots of the interaction of loneliness with spirituality, religiosity and religious participation on LLS among older adults. Although spirituality, religiosity, and religious participation have attenuated the association between loneliness and LLS, they were statistically insignificant.

## Discussion

The study examined the association between loneliness and life satisfaction among older adults and the extent to which this association was moderated by spirituality, religiosity, and religious participation. The findings revealed that older adults who are lonely had a higher likelihood of LLS than their peers who were not lonely. These findings match those in previous studies, which recommend researchers and policy makers to focus on constructive interventions to counter LLS. It includes optimizing social capital, being socially engaged, and aging in place for as long as one’s health permits [31, 73–75]. As expected, we found that spirituality, religiosity, and religious participation reduced the odds of LLS among older adults who were lonely. This mirrors the finding in a previous study in India suggestive of the positive impact of religiosity on the wellbeing among older Indians [76], and other literature indicating the positive connection between religiosity and life satisfaction [77–79].

In so far as it widens social network, and creates opportunities to interact with like-minded others, and engage in prosocial activities, religious participation may engender in older adults a sense of fulfilment and satisfaction. Being integrated within a religious group or faith-based community may mean benefits such as visits from members of these groups, this being particularly impactful for older adults who are unable to attend religious activities, thereby alleviating loneliness and improving life satisfaction [80]. Supports may also include spiritual guidance, help with meals, and rides to and from medical appointments. Such type of supports may become increasingly important for future cohorts of older adults, as family structures continue to evolve, fertility declines, and more educated adult children move away from older parents for purposes of employment. Religious participation also may translate into daily rituals of prayers and mindful meditation, which could bolster hopefulness and higher life satisfaction [81, 82].

Often interventions targeting loneliness include one-on-one intergenerational “befriending” programs in which younger volunteers reach out to older individuals identified as feeling lonely. Other interventions to increase support networks may include encouraging older adults to join support groups or participate in psychoeducational workshops and social skills training [83–85]. Although such interventions may help facilitate social interactions and in turn, network building, such strategies may not necessarily help older adults develop high-quality relationships. Loneliness related interventions also often are focused on building new networks of support without focusing any effort to nurture the perception of existing support [83, 84]. Religious engagement may help integrate individual systems of faith to bolster perceived availability of support, which is key to life satisfaction in later life [86, 87].

Multiple studies highlight the importance of religious participation in promoting life satisfaction and subjective well-being among older adults, and the association of religious attendance with life satisfaction is found to be fairly strong in comparison with other socio-economic factors [79, 88]. A micro-level study conducted in the US revealed that participation in religious education improves life satisfaction and quality of life [89]. Moreover, be it social control mechanisms of discouraging risky activities and motivating health promoting behaviors, social stimulation through social activities, sensory stimulation via activities that require thinking, processing, and memory, or emotional bandwidth through positive affect, religious participation may shape cognitive functioning [90]. Cognitive functioning, we know, determines quality of life and life expectancy for older adults.

A recent nationally representative survey [91] on religion in India revealed the importance of religious identity and religious observances for a major proportion of the respondents. Religious observances are considered vital to signify major life events such as birth, death and other milestones. The findings of the present study offers practical implications for health professionals and practitioners working with older Indians, emphasizing the importance of recognizing, respecting, and optimizing religious beliefs and their influence on wellbeing. Understanding how the faith of individuals may influence their ability to cope with loneliness may be particularly useful. Our findings also encourage developing opportunities for older adults to become more involved in offering support to others in their religious and faith-based communities as this may provide the notion of extended family [92] and improve later life wellbeing. While pharmacologic and psychiatric interventions do help older adults suffering from the physical and psychological ramifications associated with loneliness, they can produce undesirable, sometimes serious side-effects that cannot be prevented or controlled [93]. Rising age gives rise to multiple chronic conditions, which increases the risk of polypharmacy [94]. Polypharmacy or the consumption of 5 or more medications is known to significantly increase the risk of not only adverse physiological reactions, such as nausea, dizziness, and loss of appetite and behavioral side-effects including apathy [95, 96], but also compromise cognitive function which is crucial for maintaining social activity and coping with loneliness [97–99]. In these cases, social and community-level non-invasive experiences, like those accrued from religious engagement, may prove invaluable. Engaging with religious or faith-based communities can frequently improve access to social services and recreational opportunities, which can have a positive impact on one’s health and well-being both directly and indirectly [100].

That said, these findings must be interpreted with caution given that they contradict evidence in some other studies. In particular, some research finds a non-linear association between religiosity and life satisfaction [101, 102]. Moreover and importantly, religion may not always support health promoting lifestyles. For example, some religious tenets may discourage or in fact, prohibit the use of preventive health services and medical treatments (e.g., vaccinations; blood transfusion). This, in consequence, may negatively affect health and quality of life [103, 104]. What’s more is that non-compliance, in the case of certain religions, may subject older adults to judgment, potentially causing psychological distress [30, 105] which also could adversely affect wellbeing.

Apart from religious attendance and religiosity, our findings revealed that spirituality protects against LLS among older adults. Our finding mirrors that a study conducted in Italy, reiterating the importance of spirituality and religiosity as potential determinants of subjective wellbeing [32]. Spirituality, which offers a “sense of coherence” may help older adults to more positively reinterpret their lives, ultimately boosting life satisfaction [106]. Therefore, spirituality as a non-clinical factor to improve subjective wellbeing cannot be undermined. In fact, research points out the stress buffering effect of spiritual beliefs and experiences, which reduce depressive symptoms, increase hopefulness [107, 108], and improve mental health [109–111]. A recent study based on sample of Indian older adults also finds spirituality to positively affect cognitive health [112].

### Limitations of the study

While our findings contribute to research on religion and health in later life, our study has some important limitations. *First*, the study is cross-sectional in nature, which essentially precludes us from staking any definitional claims about the associations among variables of interest. A prospective, longitudinal study may illuminate how the associations among the key variables unfold over time. For instance, it may be worthwhile to differentiate the experience of temporary versus prolonged loneliness. While the former may actually encourage individuals to reconnect with others, the latter may dissuade an individual from making any effort to socially connect, which may severely compromise mental and cognitive processes necessary for a good quality of life. Longitudinal data also may permit future scholars to test the possible bidirectional relationship between loneliness and life satisfaction. *Second*, despite commonly used and fitting for large-scale, population-based survey studies, the single-item measurement of loneliness may result in underreporting given the stigma associated with being lonely [113, 114]. LASI, unfortunately, does not contain multidimensional scales of life satisfaction.

*Third*, to assess selection bias, future research should consider additional variables, such as individual pre-dispositions when looking at the modifying effect of religious attendance on loneliness and life satisfaction. Some individuals may be better equipped to garner support within their congregation and participate more actively in religious activities, simply because they are more extraverted, agreeable, and open-minded [28, 115, 116]. In fact, there is some evidence suggestive of the part personality plays in conditioning the impact religious attendance has on social support and cohesion [28]. Majority of these observations, however, are based on samples of older adults in western nations. There is a relative paucity of research assessing connections between loneliness and life satisfaction in LMICs, especially, in South Asian countries. This may reflect the commonplace assumption that in collectivistic societies, like India, older adults are well integrated socially and institutionally. However, India’s older adults are vulnerable economically and socially; and institutions, including health care, continue to lag demographic needs [117, 118]. Further, religiosity is a subjective measure and the engagement in religious participation may depend on sociocultural environment [119], especially in Indian context where religiosity plays a vital role in the lives of older adults. Considering varying sociocultural contexts may be a valuable endeavor for further research, given that it may help promote healthy aging among persons of diverse groups and backgrounds. Also, frequency of religious participation [120], and types of social or religious activities [121], have been shown to have differential impact on mental health and wellbeing of older people and should be considered in future research looking to replicate the present study. *Finally*, because our outcome measure is more frequent, the adjusted odds ratios from the logistic regression should be interpreted with caution as it may exaggerate the observed risk associations [122].

## Conclusions

The study found an independent association between loneliness and lower life satisfaction among older adults in India. It also revealed that religiosity, spirituality and religious participation moderate the association between loneliness and lower life satisfaction. These findings, which underscore the health promoting benefits of religiosity and religious engagement, may be used to build on the interaction between religious and faith-based groups and public health professionals. Religious institutions, and governmental and non-governmental agencies have often collaborated to address public health challenges [123]. This beneficial partnership should not overlook population ageing and the quality of life for older adults. That said, in India, religious diversity is a strength and a weakness. The weakness, reflecting past and present conflicts between people and institutions of varying faiths, suggests that we should focus on promoting the social, emotional, and health benefits of religious participation and religiosity.

## Data Availability

The data are available in the public domain and freely accessible from the Gateway to Global Aging Data (www.g2aging.org). The data are also available in the International Institute for Population Sciences (IIPS) data repository of Longitudinal Aging Study in India, upon request to IIPS, Mumbai, https://www.iipsindia.ac.in/content/LASI-data.

## Abbreviations

LLS: Low life satisfaction
LASI: Longitudinal aging study in India
ADL: Activities of daily living
IADL: Instrumental activities of daily living
MPCE: Monthly per capita consumption expenditure
Crude OR: Unadjusted odds ratio
Adjusted OR: Adjusted odds ratio
CI: Confidence interval

## References

[1] Han X, Yang Y, Li F et al. Adding life to years: the influence of internet use and appearance management on life satisfaction among the elderly. Soc Indic Res 2020; 1–16.

[2] Szcześniak M, Bielecka G, Madej D et al. The role of self-esteem in the relationship between loneliness and life satisfaction in late adulthood: evidence from Poland. Psychol Res Behav Manag. 10.2147/PRBM.S275902 PMC7754268 33363419

[3] Ardelt M (1997). Wisdom and life satisfaction in old age. J Gerontol B Psychol Sci Soc Sci.

[4] Mirowsky J, Ross CE. Age and depression. J Health Soc Behav 1992; 187–205. 1401846

[5] Baltes PB, Baltes MM. *Psychological perspectives on successful aging: The model of selective optimization with compensation*. 1990.

[6] Carpentieri JD, Elliott J, Brett CE (2017). Adapting to aging: older people talk about their use of selection, optimization, and compensation to maximize well-being in the context of physical decline. J Gerontol Ser B.

[7] Szcześniak M, Tu\lecka M (2020). Family functioning and life satisfaction: the mediatory role of emotional intelligence. Psychol Res Behav Manag.

[8] Gil-Lacruz M, Saz-Gil MI, Gil-Lacruz AI (2019). Benefits of older volunteering on wellbeing: an international comparison. Front Psychol.

[9] Wilson J, Musick M (1999). The effects of volunteering on the volunteer. Law Contemp Probs.

[10] Smith JL, Bihary JG, O’Connor D (2020). Impact of savoring ability on the relationship between older adults’ activity engagement and well-being. J Appl Gerontol.

[11] B\lachnio A, Buliński L. SECURING HEALTH: SOCIAL REHABILITATION AND WELLBEING IN LATE ADULTHOOD. *Acta Neuropsychol*; 11.

[12] de Jong Gierveld J, Havens B (2004). Cross-national comparisons of social isolation and loneliness: introduction and overview. Can J Aging Rev Can Vieil.

[13] Hawkley LC, Cacioppo JT (2007). Aging and loneliness: downhill quickly?. Curr Dir Psychol Sci.

[14] Loneliness and Social Isolation — Tips for Staying Connected. *National Institute on Aging*, https://www.nia.nih.gov/health/loneliness-and-social-isolation-tips-staying-connected (accessed 2 April 2023).

[15] Perissinotto CM, Covinsky KE (2014). Living alone, socially isolated or lonely—what are we measuring?. J Gen Intern Med.

[16] Coyle CE, Dugan E (2012). Social isolation, loneliness and health among older adults. J Aging Health.

[17] Schrempft S, Jackowska M, Hamer M (2019). Associations between social isolation, loneliness, and objective physical activity in older men and women. BMC Public Health.

[18] Cacioppo JT, Cacioppo S (2018). The growing problem of loneliness. The Lancet.

[19] Donovan NJ, Blazer D (2020). Social isolation and loneliness in older adults: review and commentary of a National Academies report. Am J Geriatr Psychiatry.

[20] Fakoya OA, McCorry NK, Donnelly M (2020). Loneliness and social isolation interventions for older adults: a scoping review of reviews. BMC Public Health.

[21] DiNapoli EA, Wu B, Scogin F (2014). Social isolation and cognitive function in Appalachian older adults. Res Aging.

[22] Heape A (2021). Loneliness and social isolation in older adults: the Effects of a pandemic. Perspect ASHA Spec Interest Groups.

[23] Nicholson NR (2012). A review of social isolation: an important but underassessed condition in older adults. J Prim Prev.

[24] Carr D, Moorman SM. Social relations and aging. Handbook of sociology of aging. Springer, 2011, 145–60.

[25] Idler EL, Discussion (2003). Gender differences in self-rated health, in mortality, and in the relationship between the two. Gerontologist.

[26] Krause N (1997). Religion, aging, and health: current status and future prospects. J Gerontol B Psychol Sci Soc Sci.

[27] Levin JS, Chatters LM (1998). Religion, health, and psychological well-being in older adults: findings from three national surveys. J Aging Health.

[28] Bradley E. Religious involvement and social resources: evidence from the data set” Americans’ changing lives”. J Sci Study Relig 1995; 259–67.

[29] Ellison CG, George LK. Religious involvement, social ties, and social support in a southeastern community. J Sci Study Relig 1994; 46–61.

[30] Krause N (2002). Church-based social support and health in old age: exploring variations by race. J Gerontol B Psychol Sci Soc Sci.

[31] Ponce MSH, Rosas RPE, Lorca MBF (2014). Social capital, social participation and life satisfaction among chilean older adults. Rev Saude Publica.

[32] Villani D, Sorgente A, Iannello P (2019). The role of spirituality and religiosity in subjective well-being of individuals with different religious status. Front Psychol.

[33] Yoon DP, Lee E-KO. Religiousness/spirituality and subjective well-being among rural elderly whites, African Americans, and native Americans. Diversity and aging in the Social Environment. Routledge, 2016, 191–211.

[34] Vishkin A, Bigman YE, Porat R (2016). God rest our hearts: religiosity and cognitive reappraisal. Emotion.

[35] Vishkin A, Ben-Nun Bloom P, Tamir M (2019). Always look on the bright side of life: religiosity, emotion regulation and well-being in a jewish and christian sample. J Happiness Stud.

[36] Bożek A, Nowak PF, Blukacz M (2020). The relationship between spirituality, health-related behavior, and psychological well-being. Front Psychol.

[37] David R, Singh S, Ribeiro N (2022). Does Spiritual Influence Happiness Acad Performance? Religions.

[38] Lambert NM, Fincham FD, Braithwaite SR (2009). Can prayer increase gratitude?. Psychol Relig Spiritual.

[39] KURNAZ MF, Esra T, GÜNAYDIN HA (2020). Relationship between self-esteem and life satisfaction: a meta-analysis study. Res Educ Psychol.

[40] UN. *World population prospects 2019*. 2019.

[41] Muhammad T (2022). The role of religiosity and religious participation in the relationship between depressive symptoms and cognitive impairment among older indian adults. Sci Rep.

[42] Chakravorty S, Goli S, James KS. Family Demography in India: emerging patterns and its Challenges. SAGE Open. 2021;11. 10.1177/21582440211008178. Epub ahead of print.

[43] Goli S, Bheemeshwar Reddy A, James KS (2019). Economic independence and social security among India’s elderly. Econ Polit Wkly.

[44] Mitchell T. 1. Why do levels of religious observance vary by age and country? *Pew Research Center’s Religion* & *Public Life Project*, https://www.pewresearch.org/religion/2018/06/13/why-do-levels-of-religious-observance-vary-by-age-and-country/ (2018, accessed 21 December 2022).

[45] Zimmer Z, Jagger C, Chiu C-T (2016). Spirituality, religiosity, aging and health in global perspective: a review. SSM-Popul Health.

[46] Villani D, Sorgente A, Iannello P et al. The Role of Spirituality and Religiosity in Subjective Well-Being of Individuals With Different Religious Status. *Front Psychol*; 10, https://www.frontiersin.org/articles/10.3389/fpsyg.2019.01525 (2019, accessed 2 April 2023). 10.3389/fpsyg.2019.01525 PMC6630357 31354566

[47] Iannello NM, Hardy SA, Musso P (2019). Spirituality and ethnocultural empathy among italian adolescents: the mediating role of religious identity formation processes. Psychol Relig Spiritual.

[48] Zinnbauer BJ, Pargament KI, Scott AB (1999). The emerging meanings of religiousness and spirituality: problems and prospects. J Pers.

[49] Worthington EL, Hook JN, Davis DE (2011). Religion and spirituality. J Clin Psychol.

[50] Rojas M, Watkins-Fassler K (2022). Religious practice and life satisfaction: a Domains-of-life Approach. J Happiness Stud.

[51] Ellison CG (1991). Religious involvement and subjective well-being. J Health Soc Behav.

[52] International Institute for Population Sciences (IIPS)., NPHCE, MoHFW HTHCS of PH (HSPH) and the U of SC (USC). *Longitudinal Ageing Study in India (LASI) Wave 1, 2017-18, India Report*. Mumbai., 2020.

[53] Diener ED, Emmons RA, Larsen RJ (1985). The satisfaction with life scale. J Pers Assess.

[54] Maynard M, Andrade L, Packull-McCormick S (2018). Food insecurity and mental health among females in high-income countries. Int J Environ Res Public Health.

[55] Chou KL, Ho AHY, Chi I (2006). Living alone and depression in chinese older adults. Aging Ment Health.

[56] Zhang Z, Li LW, Xu H (2019). Does widowhood affect cognitive function among chinese older adults?. SSM - Popul Health.

[57] Lee EE, Depp C, Palmer BW (2019). High prevalence and adverse health effects of loneliness in community-dwelling adults across the lifespan: role of wisdom as a protective factor. Int Psychogeriatr.

[58] Underwood L (2006). Ordinary spiritual experience: qualitative research, interpretive guidelines, and population distribution for the daily spiritual experience scale. Arch Psychol Relig.

[59] Underwood LG, Teresi JA (2002). The daily spiritual experience scale: development, theoretical description, reliability, exploratory factor analysis, and preliminary construct validity using health-related data. Ann Behav Med.

[60] Kimura M, de Oliveira AL, Mishima LS (2012). Cultural adaptation and validation of the Underwood’s daily spiritual experience scale-brazilian version. Rev Esc Enferm USP.

[61] Loustalot F, Wyatt SB, Sims M (2011). Psychometric testing of the daily spiritual experiences scale among African Americans in the Jackson Heart Study. J Relig Health.

[62] Jayakumar T, Verma A (2021). Indic Religiosity Scale: developing and validating an indian religiosity scale. J Manag Spiritual Relig.

[63] Svob C, Wong LY, Gameroff MJ (2019). Understanding self-reported importance of religion/spirituality in a north american sample of individuals at risk for familial depression: a principal component analysis. PLoS ONE.

[64] Idler EL (1987). Religious involvement and the health of the elderly: some hypotheses and an initial test. Soc Forces.

[65] Benjamins MR, Musick MA, Gold DT (2003). Age-related declines in activity level: the relationship between chronic illness and religious activities. J Gerontol B Psychol Sci Soc Sci.

[66] Idler EL, Kasl SV (1997). Religion among disabled and nondisabled persons I: cross-sectional patterns in health practices, social activities, and well-being Ellen. J Gerontol B Psychol Sci Soc Sci.

[67] Kelley-Moore JA, Ferraro KF (2001). Functional limitations and religious service attendance in later life: barrier and/or benefit mechanism?. J Gerontol B Psychol Sci Soc Sci.

[68] Srivastava S, Dristhi JVJK (2021). Interaction of physical activity on the related measures association of obesity- ­ with multimorbidity among older adults: a population- ­ based cross- ­ sectional study in India. BMJ Open Epub ahead of print.

[69] Muhammad T, Hossain B, Das A et al. Relationship between handgrip strength and self-reported functional difficulties among older indian adults: the role of self-rated health. Exp Gerontol 2022; 111833. 10.1016/j.exger.2022.111833 35577266

[70] Hu P, Wang S, Lee J (2017). Socioeconomic gradients of cardiovascular risk factors in China and India: results from the China health and retirement longitudinal study and longitudinal aging study in India. Int J Public Health.

[71] Borooah VK (2018). Caste, religion, and health outcomes in India, 2004–14. Econ Polit Wkly.

[72] Nayar KR (2007). Social exclusion, caste & health: a review based on the social determinants framework. Indian J Med Res.

[73] X B (2010). Social support, loneliness and life satisfaction in older adults living alone. Gerontologist.

[74] Tümer A, Dönmez S, Gümüşsoy S et al. The relationship among aging in place, loneliness, and life satisfaction in the elderly in Turkey. Perspect Psychiatr Care 2021; 1–8. 10.1111/ppc.12855 34018200

[75] Annalisa D, Silvia M, Letizia TM. *Social activities, loneliness and life satisfaction in old age: a time use study* 10/2020, Padova, 2020.

[76] Chokkanathan S (2013). Religiosity and well-being of older adults in Chennai, India. Aging Ment Health.

[77] Sharif SP, Amiri M, Allen KA (2021). Attachment: the mediating role of hope, religiosity, and life satisfaction in older adults. Health Qual Life Outcomes.

[78] Beach V. *Religiosity and prayer in relation to health and life satisfaction in older adults*. 2016.

[79] Barkan SE, Greenwood SF (2003). Religious attendance and subjective well-being among older Americans: evidence from the general social survey. Rev Relig Res.

[80] Cnaan RA, Boddie SC, Kang JJ (2005). Religious congregations as social services providers for older adults. J Gerontol Soc Work.

[81] Lazar A (2015). The relation between prayer type and life satisfaction in religious jewish men and women: the moderating Effects of prayer duration and belief in prayer. Int J Psychol Relig.

[82] Upenieks L (2021). Never more than I can handle? A longitudinal consideration of racial differences in trust-based prayer expectancies of god and satisfaction in later life. J Relig Spiritual Aging.

[83] Lakey B, Lutz CJ. Social support and preventive and therapeutic interventions. In: Pierce GR, Sarason BR, Sarason IG, editors *Handbook of Social Support and the family*. Boston, MA: Springer US, pp. 435–65.

[84] Brand EF, Lakey B, Berman S (1995). A preventive, psychoeducational approach to increase perceived social support. Am J Community Psychol.

[85] Mann F, Bone JK, Lloyd-Evans B (2017). A life less lonely: the state of the art in interventions to reduce loneliness in people with mental health problems. Soc Psychiatry Psychiatr Epidemiol.

[86] Shen Y, Yeatts DE (2013). Social support and life satisfaction among older adults in China: family-based support versus community-based support. Int J Aging Hum Dev.

[87] Moghadam K, Mansour-Ghanaei R, Esmaeilpour-Bandboni M (2020). Investigating the relationship between social support and quality of life in the elderly. J Educ Health Promot.

[88] Hill TD, Burdette AM, Angel JL (2006). Religious attendance and cognitive functioning among older Mexican Americans. J Gerontol - Ser B Psychol Sci Soc Sci.

[89] Maxwell DD, Edd IC (1996). Participation in religious education and life satisfaction among older adults. J Relig Gerontol.

[90] Henderson AK, Walsemann KM, Ailshire JA (2022). Religious involvement and cognitive functioning at the intersection of race–ethnicity and gender among midlife and older adults. J Gerontol Ser B.

[91] Pew Research Center. Religion in India: Tolerance and Segregation.

[92] Koenig HG (2004). Religion, spirituality, and medicine: research findings and implications for clinical practice. South Med J.

[93] White N, Leurent B, Lord K (2017). The management of behavioural and psychological symptoms of dementia in the acute general medical hospital: a longitudinal cohort study. Int J Geriatr Psychiatry.

[94] Kim H-A, Shin J-Y, Kim M-H (2014). Prevalence and predictors of polypharmacy among korean elderly. PLoS ONE.

[95] Hofer-Dückelmann C. Gender and polypharmacotherapy in the elderly: a clinical challenge. Handb Exp Pharmacol 2012; 169–82. 10.1007/978-3-642-30726-3_9 23027451

[96] Chiang-Hanisko L, Tan J-Y, Chiang L-C (2014). [Polypharmacy issues in older adults]. Hu Li Za Zhi.

[97] Assari S, Wisseh C, Saqib M (2020). Polypharmacy is Associated with lower memory function in african american older adults. Brain Sci.

[98] Khezrian M, McNeil CJ, Myint PK (2019). The association between polypharmacy and late life deficits in cognitive, physical and emotional capability: a cohort study. Int J Clin Pharm.

[99] Oyarzun-Gonzalez XA, Taylor KC, Myers SR (2015). Cognitive decline and Polypharmacy in an Elderly Population. J Am Geriatr Soc.

[100] South J, Higgins TJ, Woodall J (2008). Can social prescribing provide the missing link?. Prim Health Care Res Dev.

[101] Okulicz-kozaryn A. Religiosity and life satisfaction across nations. Ment Health Relig Cult 2009; 155–69.

[102] Yeniaras V, Akarsu TN (2017). Religiosity and life satisfaction: a multi-dimensional approach. J Happiness Stud.

[103] Ellison CG, Taylor RJ. Turning to prayer: Social and situational antecedents of religious coping among African Americans. Rev Relig Res 1996; 111–31.

[104] Neighbors HW, Musick MA, Williams DR (1998). The african american minister as a source of help for serious personal crises: Bridge or barrier to mental health care?. Health Educ Behav.

[105] Krause N, Chatters LM, Meltzer T et al. Negative interaction in the church: insights from focus groups with older adults. Rev Relig Res 2000; 510–33.

[106] Cowlishaw S, Niele S, Teshuva K (2013). Older adults’ spirituality and life satisfaction: a longitudinal test of social support and sense of coherence as mediating mechanisms. Ageing Soc.

[107] Ozdemir S, Palframan JT, Sever M et al. Workplace spirituality as a mediator between organizational trust and thriving at work. Vision 2022; 09722629221101156.

[108] Kim SN, Lee SB (2013). Spiritual well-being, social support, life satisfaction and depression in the community dwelling elderly. J East-West Nurs Res.

[109] Koenig HG (2010). Spirituality and mental health. Int J Appl Psychoanal Stud.

[110] Wu L-F, Koo M (2016). Randomized controlled trial of a six-week spiritual reminiscence intervention on hope, life satisfaction, and spiritual well-being in elderly with mild and moderate dementia. Int J Geriatr Psychiatry.

[111] Taghiabadi M, Kavosi A, Mirhafez SR (2017). The association between death anxiety with spiritual experiences and life satisfaction in elderly people. Electron Physician.

[112] Barman P, Saha A, Dakua M et al. Does the intensity of religiosity and spirituality in later life improve mental well-being? Evidence from India. J Relig Spiritual Aging 2022; 1–21.

[113] Pinquart M, Sorensen S (2001). Influences on loneliness in older adults: a meta-analysis. Basic Appl Soc Psychol.

[114] Shiovitz-Ezra S, Ayalon L (2012). Use of direct versus indirect approaches to measure loneliness in later life. Res Aging.

[115] Clark EM, Holt CL, Wang MQ (2017). Which personality traits moderate the relationship between religious capital and depressive symptomology in a national sample of African Americans?. J Black Psychol.

[116] Hill TD, Burdette AM, Idler EL. Religious involvement, health status, and mortality risk. Handbook of sociology of aging. Springer, 2011, 533–46.

[117] Kasthuri A (2018). Challenges to Healthcare in India - The Five A’s. Indian J Community Med Off Publ Indian Assoc Prev Soc Med.

[118] Sudharsanan N, Bloom DE, Sudharsanan N. The demography of aging in low-and middle-income countries: chronological versus functional perspectives. In: *Future directions for the demography of aging: Proceedings of a workshop*. 2018, pp. 309–338.

[119] Malone J, Dadswell A (2018). The role of religion, spirituality and/or belief in positive ageing for older adults. Geriatrics.

[120] Chen H, Cheal K, McDonel Herr EC (2007). Religious participation as a predictor of mental health status and treatment outcomes in older persons. Int J Geriatr Psychiatry J Psychiatry Late Life Allied Sci.

[121] Gautam R, Saito T, Kai I (2007). Leisure and religious activity participation and mental health: gender analysis of older adults in Nepal. BMC Public Health.

[122] Zhang J, Kai FY (1998). What’s the relative risk?: A method of correcting the odds ratio in cohort studies of common outcomes. JAMA.

[123] Levin J (2014). Faith-based partnerships for population health: challenges, initiatives, and prospects. Public Health Rep.

